# Musculoskeletal Infection: The Great Mimickers on Imaging

**DOI:** 10.3390/jcm13185424

**Published:** 2024-09-13

**Authors:** Michail E. Klontzas, Evangelia E. Vassalou, Konstantinos Spanakis, Kalliopi Alpantaki, Apostolos H. Karantanas

**Affiliations:** 1 Department of Medical Imaging, University Hospital of Heraklion, 71110 Crete, Greece; 2 Computational BioMedicine Laboratory, Institute of Computer Science, Foundation for Research and Technology-Hellas (FORTH), 70013 Heraklion, Greece; 3 Department of Radiology, School of Medicine, University of Crete, 71003 Heraklion, Greece; 4 Department of Orthopaedics and Trauma Surgery, “Venizeleion” General Hospital of Heraklion, 71409 Crete, Greece

**Keywords:** musculoskeletal, infection, mimicker, differential, septic, osteomyelitis, septic arthritis, spondylodiscitis

## Abstract

A series of conditions can mimic musculoskeletal infections on imaging, complicating their diagnosis and affecting the treatment. Depending on the anatomical location, different conditions can manifest with clinical and imaging findings that mimic infections. Herein we present a wide spectrum of the musculoskeletal disorders of the axial skeleton, long bones, peripheral joints, and soft tissue that may manifest as infectious processes, and we focus on the potential mimics of osteomyelitis, septic arthritis, and infectious spondylodiscitis that are common in clinical practice. We present the typical imaging characteristics of each musculoskeletal infection, followed by mimicking conditions.

## 1. Introduction

The clinical and imaging manifestations of musculoskeletal infections are variable, depending on the underlying pathogen as well as on host-related factors such as the patient’s immune status, the site of the involvement, and the duration of the infection. Furthermore, a variety of non-infectious diseases such as tumors, trauma, and autoimmune or degenerative conditions may mimic septic disorders, making the diagnosis challenging [1]. Multidisciplinary approaches and the use of various diagnostic imaging techniques are often required [2]. Considering musculoskeletal infectious processes, the main differentials for the axial skeleton, long bones, and peripheral joints include inflammatory conditions (in the context of aseptic spondylodiscitis, rheumatoid arthritis (RA), SAPHO syndrome and non-infectious sacroiliitis), degenerative disorders, crystal deposition, trauma, neoplasms, and neuropathic arthropathy. Regarding muscles and soft tissues, the main entities that may mimic infection include non-infectious subcutaneous edema and fasciitis, muscle denervation and trauma, neoplasms, and post-treatment changes. Imaging plays a fundamental role in the diagnosis of infection, evaluating the extent of the disease and the associated complications, planning biopsy sites, and monitoring treatment responses. This is of the utmost importance since spinal infections are not necessarily accompanied by increased C-reactive protein (CRP) or fever, which are expected in other types of infection, rendering imaging extremely important in differentiating from non-infectious causes. A variety of imaging modalities, including plain radiography, ultrasound, computed tomography (CT), magnetic resonance (MR) imaging, and functional studies such as nuclear scintigraphy (such as indium 111 white blood cell scan), and positron emission tomography (PET), are available today [3]. The purpose of this paper is to familiarize clinicians with the lesions that may mimic musculoskeletal infections and to explore imaging techniques that are employed in the process of a differential diagnosis. Herein we present a wide spectrum of the musculoskeletal disorders of the axial skeleton, long bones, peripheral joints, and soft tissue that may manifest as an infectious process, and we focus on the potential mimics of osteomyelitis, septic arthritis, and infectious spondylodiscitis that are common in clinical practice. We present the typical imaging characteristics of each musculoskeletal infection, followed by mimicking conditions.

## 2. Spine and Sacroiliac Joints

### 2.1. Non-Infectious Discitis and Spondylodiscitis

**Figure 1:**
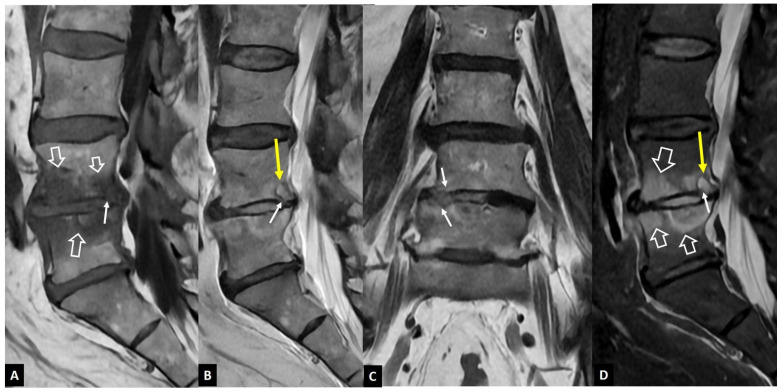
A 73-year-old male patient with *Staphylococcus aureus* infectious spondylodiscitis. Sagittal T1-w (**A**) and T2-w (**B**), coronal T2-w (**C**), and sagittal STIR (**D**) MR images showing the bone marrow edema at L4 and L5 (open arrows), the endplate cortical disruption (small arrows), and the abnormally high signal within the disc on T2-w and STIR images. A small intraosseous abscess formation is also seen (long arrows).

The distinction between the inflammatory versus the infectious involvement of the disc is of major clinical importance. The accurate diagnosis of infectious spondylodiscitis requires the typical imaging findings and the isolation of the microorganism, either in the blood or in aspirated specimens. MR imaging is considered the imaging method with the highest diagnostic accuracy. The characteristic MR imaging findings in early infectious spondylodiscitis include hypo- or isointensity on T1-weighted (w) and hyperintense signal intensity on fat-suppressed images, in the subchondral endplates and the intervening disc (Figure 1). The signal alterations usually initiate in the anterior aspect of the vertebral body, affecting one or more spinal segments, and they can be unilateral at the early stage, and the cortical disruption of the endplates is an important and specific finding. The contrast enhancement of the vertebral endplate may show different patterns, such as diffuse, patchy, or linear, parallel to the endplates [4].

Various non-inflammatory disorders may involve the disc and the epiphyseal plates and simulate infections in the early phase. The most commonly encountered are discogenic degenerative changes (MODIC type) and, less commonly, aseptic discitis, also known as “Andersson’s lesion” in the context of axial spondyloarthropathy (axSpA), synovitis-acne-pustulosis-hyperostosis-osteitis (SAPHO) syndrome, crystal deposition disease, and destructive spondyloarthropathy in patients undergoing long-term hemodialysis. Post-operative changes and recent trauma may rarely mimic infection.

#### 2.1.1. MODIC I Changes

**Figure 2:**
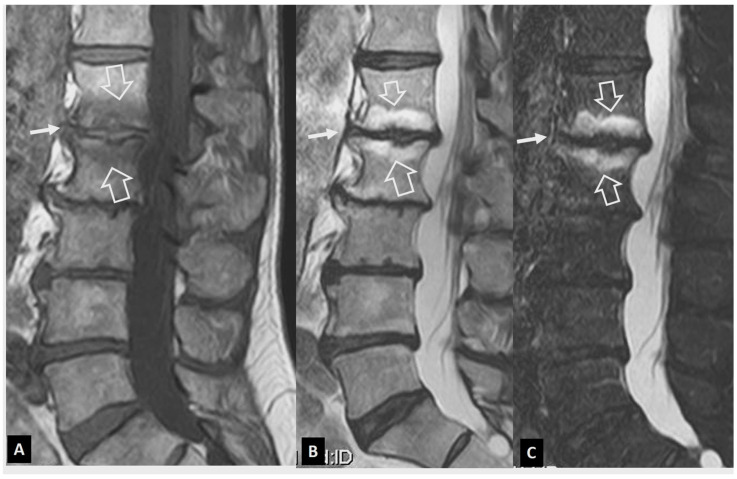
A 66-year-old male patient with a long history of scoliosis and non-specific low back pain. Sagittal T1-w (**A**), T2-w (**B**), and STIR (**C**) MR images show a dehydrated L1–L2 disc, with severe height reduction (thin arrows) and associated bone marrow edema (open arrows) in keeping with MODIC type I changes.

**Figure 3:**
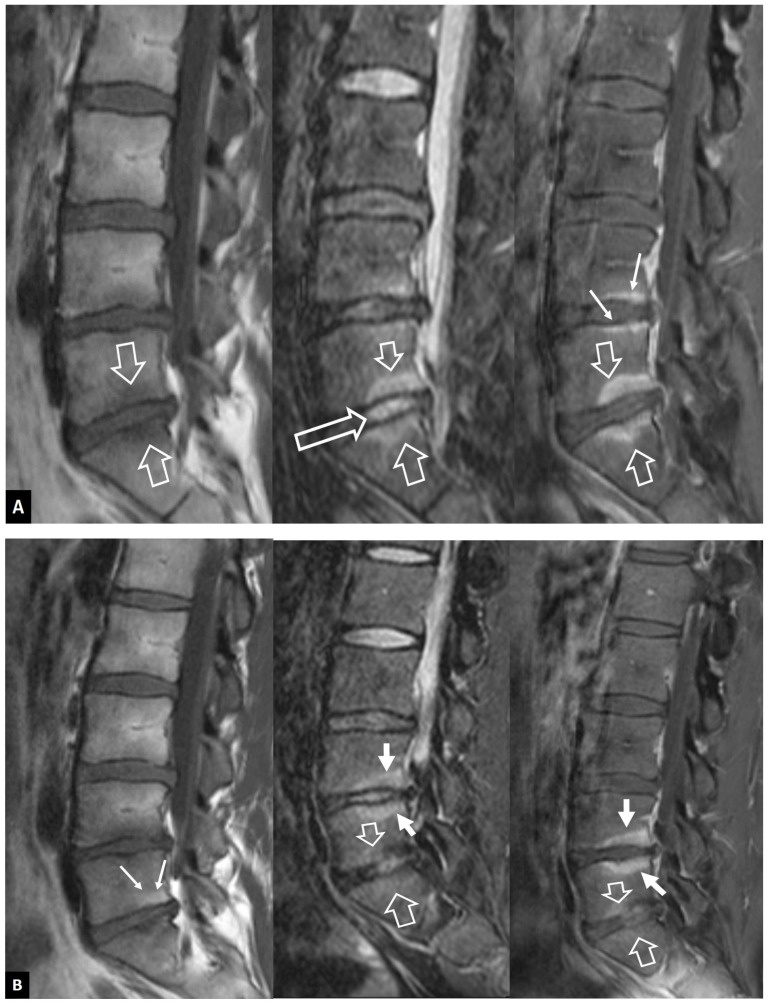

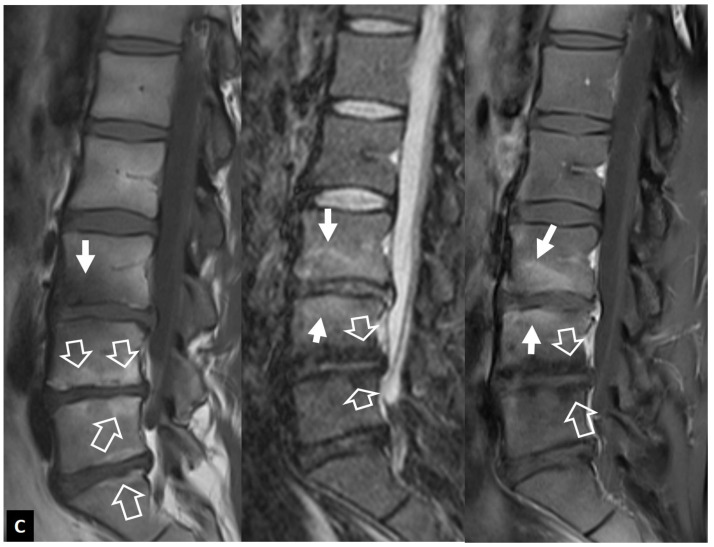
The evolution of MODIC I changes in a 31-year-old female patient with non-specific low back pain. (**A**) MR imaging examination in October 2015. Sagittal T1-w (left), STIR (center), and fat-suppressed contrast-enhanced T1-w MR images (right) showing the MODIC I changes at the L5-S1 intervertebral endplates, returning low T1 signals, high on STIR, and with enhancement on contrast-enhanced images (open arrows). Thin linear enhancement is also shown at the L4–L5 intervertebral disc space (thin arrows). Of note, the L5-S1 disc is not totally degenerated (long open arrow on STIR). (**B**) MR imaging examination in May 2016. Sagittal T1-w (left), STIR (center), and fat-suppressed contrast-enhanced T1-w MR images (right) show that the MODIC I changes at the L5-S1 intervertebral endplates have decreased (open arrows) and progressed to MODIC II with fatty replacement (thin arrows on the left image). The MODIC I changes at the L4–L5 endplates have increased (thick arrows). (**C**) MR imaging examination in March 2021. (**A**) Sagittal T1-w (left), STIR (center), and fat-suppressed contrast-enhanced T1-w (right) MR images showing the transformation of MODIC I to MODIC II changes at the L4–L5 and L5-S1 intervertebral endplates, returning high T1 signals, low on STIR, and with a lack of enhancement on contrast-enhanced images (open arrows). New MODIC I changes are located at the L3–L4 endplates (thick arrows).

**Figure 4:**
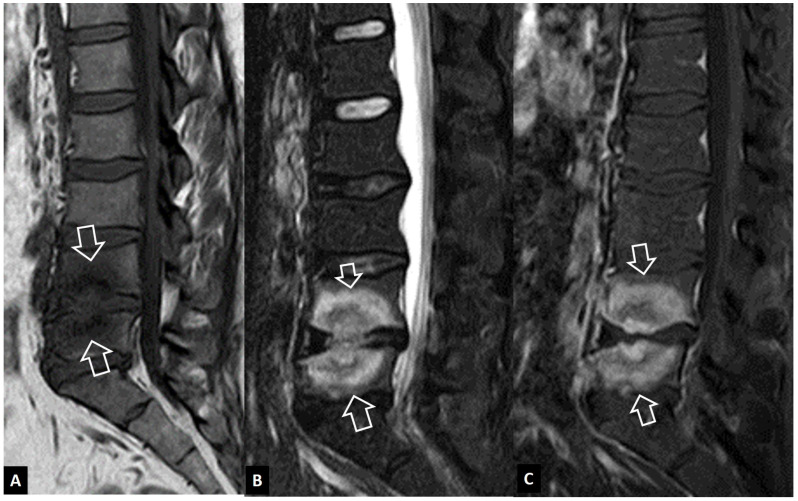
Aseptic spondylodiscitis (Andersson’s lesion) in a 38-year-old male patient with a history of 7-year low back pain. (**A**) Sagittal T1-w MR image showing low signal intensity at the endplates of the L4–L5 disc space (arrows). (**B**) Sagittal STIR MR image showing the bone marrow edema with hemispheric configuration (arrows). (**C**) Sagittal fat-suppressed contrast-enhanced T1-w MR image showing enhancement of the bone marrow edema (arrows) but no enhancement in the disc.

It is more than three decades since Modic et al. described the MR imaging findings of MODIC I–III changes, which have been attributed to traumatic injuries to the vertebral endplates [5], localized inflammation [6], and even low-grade bacterial infection [7]. MODIC I endplate changes show low T1-w and high T2-w/STIR signals within the bone marrow adjacent to the endplates. MODIC I changes are linked to non-specific low back pain and may increase in size, progress to MODIC II changes, or even regress over time [8] (Figure 2 and Figure 3). Enhancement of the bone marrow can occur in both infectious spondylodiscitis and MODIC I changes (Figure 3). Thus, the signal of the disc is the cornerstone contributing to accurate diagnosis. A low T2-w/STIR disc signal is typically seen in MODIC I changes. In the absence of the typical degeneration of the disc accompanied by disc height reduction, the discrimination with infection may be demanding, and short-interval follow-up MR imaging may be required (Figure 4). An irregular yet intact endplate contour shows high diagnostic accuracy for the differentiation between MODIC I changes and early infectious spondylodiscitis [9]. Recently, the value of diffusion-w MR imaging has been addressed, by showing hyperintensity in all patients with infectious spondylodiscitis and hypointensity in those with MODIC I changes [10].

#### 2.1.2. Aseptic Spondylodiscitis

Aseptic spondylodiscitis, also known as an “Andersson’s lesion”, is an uncommon manifestation of axSpA, first described by Andersson in 1937 [11]. It represents inflammatory enthesopathy at the interface between the disc and the endplates, and thus it involves the disc and adjacent vertebral bone marrow in the absence of infection, degeneration, or trauma [12]. The incidence of Andersson’s lesions is low on plain radiographs but high on MR imaging [13]. They are observed in 33% of patients with axSpA with spinal involvement on MR imaging, and its presence has a specificity of 59% [14].

**Figure 5:**
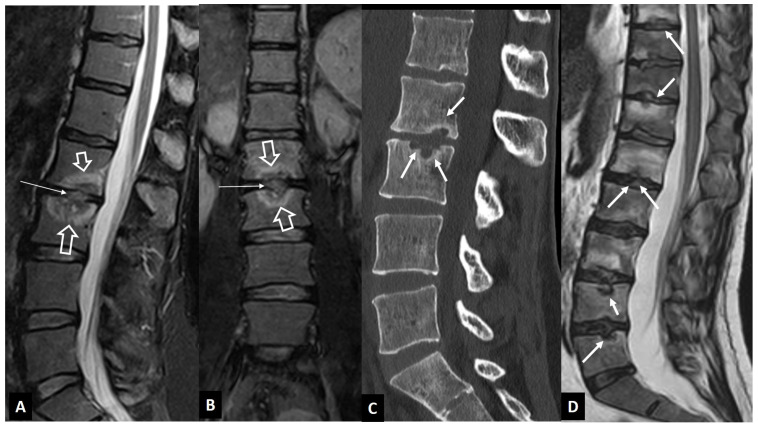
Andersson’s lesions. Sagittal STIR (**A**), coronal STIR (**B**), MR images, and sagittal CT reconstruction (**C**) in a 35-year-old male with axSpA, showing the erosions of the endplates (arrows) and the bone marrow edema (open arrows). A high signal is shown in the center of discs (thin long arrows), simulating infectious discitis. (**D**) Sagittal T2-w MR image in a 43-year-old male with axSpA showing Andersson’s lesions at multiple levels (arrows).

Andersson’s lesions are usually located in the thoracolumbar spine [15,16]. Typical findings on plain radiographs include disc space narrowing, erosions of the vertebral endplate, and sclerosis of the adjacent cancellous bone [13]. MR imaging shows high signal intensity in the disc and endplates on STIR and fat-suppressed contrast-enhanced T1-w images [17,18]. The bone marrow edema has a characteristic hemispheric configuration (Figure 4 and Figure 5). Erosions and surrounding sclerosis are better shown on CT (Figure 5C). One or more levels may be involved.

#### 2.1.3. SAPHO Syndrome

**Figure 6:**
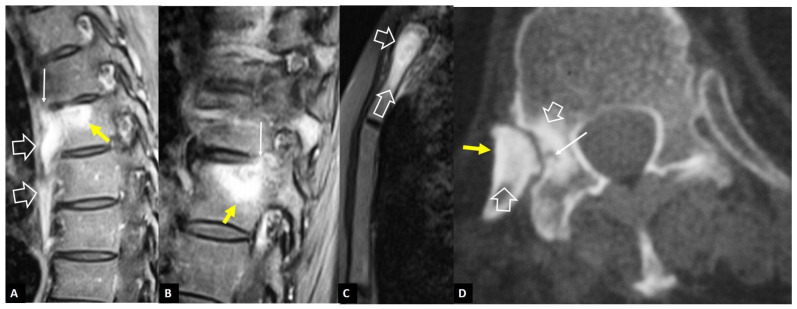
SAPHO syndrome in a 39-year-old female patient with acne, postulosis, and chronic sternal and back pain. Parasagittal fat-suppressed contrast-enhanced T1-w MR images (**A**,**B**) showing enhancing bone marrow edema in two different levels (yellow arrows) with early disc involvement (thin arrows) and soft-tissue inflammatory changes anterior to the thoracic spine (open arrows). (**C**) Sagittal STIR MR image showing the bone marrow edema in the manubrium of the sternum in keeping with osteitis (open arrows). (**D**) Axial CT showing hyperostosis of the right rib (yellow arrow), osteitis (open arrows), and erosion (thin arrow).

SAPHO syndrome belongs to a group of skin and bone disorders characterized by synovitis, acne, postulosis, hyperostosis, and osteitis, and the thoracolumbar spine is the second most frequent location [19]. Lesions within this syndrome include endplate erosions, mostly anteriorly, subchondral sclerosis, and osseous fusion. In the active phase, MR imaging shows bone marrow edema, often with soft-tissue inflammation (Figure 6). The intervertebral disc, as a rule, is spared. Other typical locations, such as the sternum and CT findings of hyperostosis and osteosclerosis, help to make the correct diagnosis (Figure 6).

As with other mimics of infection, FDG-PET shows a non-specific uptake and is, therefore, not helpful in the initial diagnosis. However, it may have a role in monitoring the response to therapy [4].

#### 2.1.4. Destructive Spondyloarthropathy

**Figure 7:**
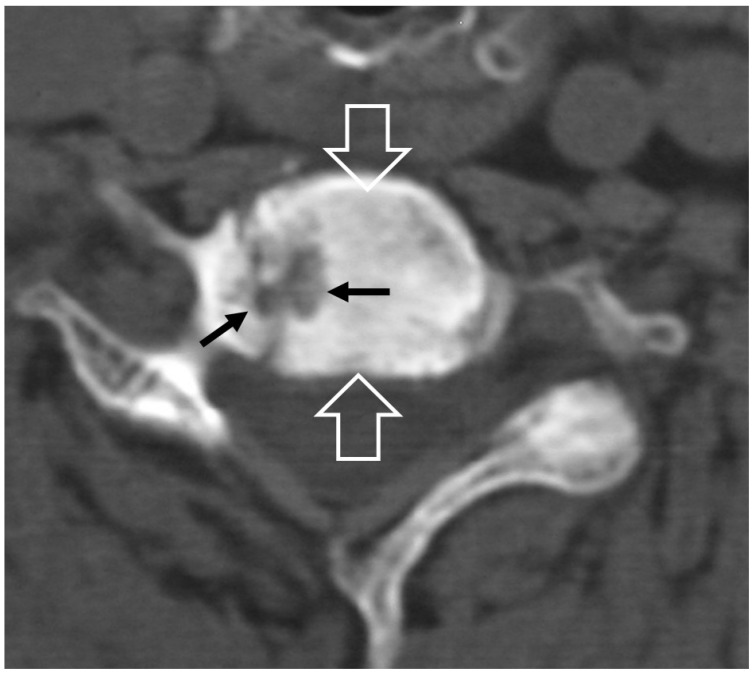
A 65-year-old chronic renal failure female patient, undergoing hemodialysis for 12 years. She has been complaining of neck pain for 6 months. Axial CT showing osteolytic lesions (arrows) and osteosclerosis (open arrows) at the C6 vertebra.

The term “destructive spondyloarthropathy” (DS) was introduced by Kuntz et al., for patients undergoing chronic hemodialysis [20]. It is primarily the result of the extensive deposition of beta-2 microglobulin (amyloid-like tissue) within the intervertebral discs and is seen in chronic renal failure patients with a long duration of hemodialysis. This disorder is characterized by rapidly progressive imaging findings such as loss of the intervertebral disc space, erosions in the endplates, and sclerosis, simulating infectious spondylodiscitis [21]. Plain radiographs and CT show non-specific findings such as erosions and cyst formation adjacent to the endplates at single or multiple levels (Figure 7). Amyloid deposits return a low signal on all pulse sequences. The disc signal on T2-w/STIR images may occasionally be high but not as high as in infectious spondylodiscitis. Vertebral body collapse and disc space narrowing may be also seen. Since chronic renal failure patients undergoing hemodialysis are at increased risk for infection, imaging is important for the differential diagnosis.

#### 2.1.5. Crystal Deposition

**Figure 8:**
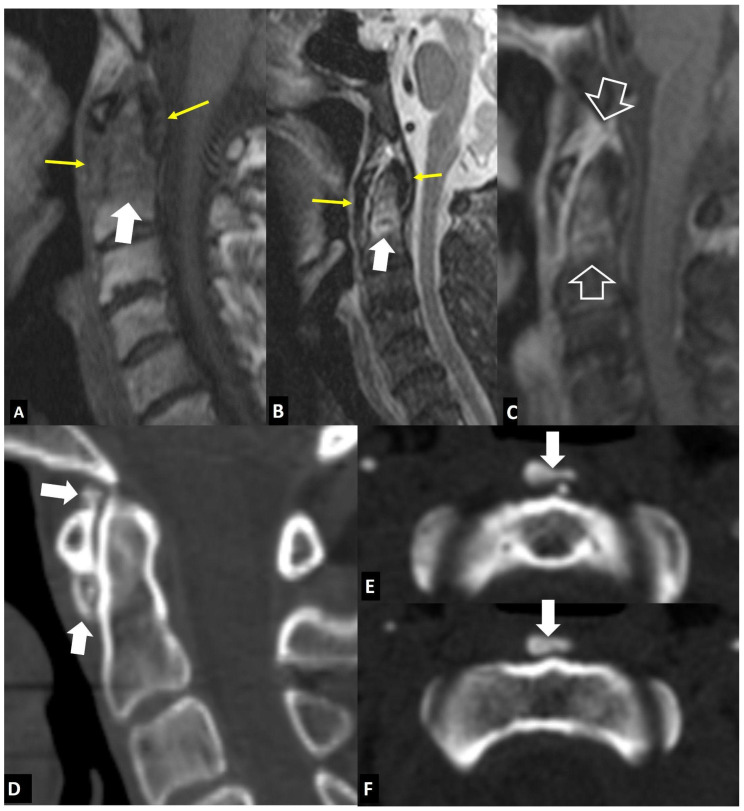
Hydroxyapatite crystal deposition disease. Sagittal T1-w (**A**), STIR (**B**), and fat-suppressed contrast-enhanced T1-w (**C**) MR images showing bone marrow edema (arrows) in the C2 vertebra, abnormal paraspinal tissue, returning intermediate signals on T1-w and low on T2-w images (thin arrows), and enhancement of both the tissue and bone marrow edema (open arrows), in a 76-year-old female patient with intense neck pain. Sagittal (**D**) and axial (**E**,**F**) CT images in a 39-year-old female patient with intense neck pain show to better advantage the crystal deposition (arrows).

Hydroxyapatite crystal deposition disease (HADD) or “Calcium Apatite Deposition Disease” or “Basic Calcium Phosphate Deposition” is a disease of uncertain etiology characterized by periarticular and intra-articular calcium deposits. An acute attack of the longus colli muscle is rare and affects patients who are between 30 and 60 years, without gender predilection [22]. The most frequent presentation is severe neck pain with rapid onset, neck stiffness, dysphagia, and headache. Fever with increased inflammatory markers may mimic clinically infectious spondylitis, retropharyngeal abscess, or meningitis. MR imaging shows prevertebral soft-tissue changes and often reactive bone marrow edema, usually in the upper cervical spine (Figure 8). CT is more sensitive for the detection of calcification and thus a correct diagnosis can be better made by combining CT and MR imaging [23].

Calcium pyrophosphate deposition disease (CPPD), previously known as “pseudogout”, is an inflammatory arthropathy characterized by the presence of calcium pyrophosphate crystals in articular or periarticular tissues [24]. Spinal CPPD is uncommon and may mimic more common spine disorders, including infection [25]. CPPD occurs in practically all vertebral structures and can give rise to acute attacks. Massive CPPD may cause myelopathy, radicular pain, or cauda equina syndrome.

**Figure 9:**
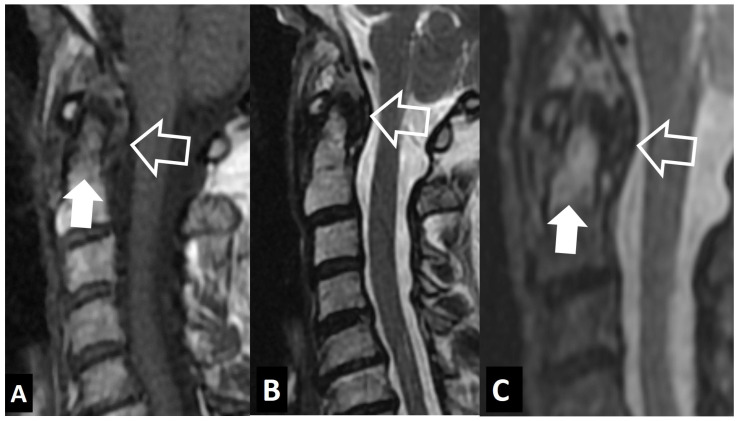

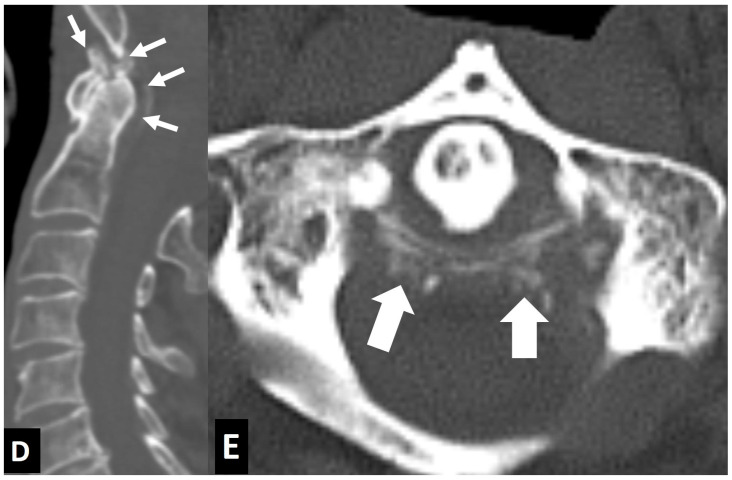
“Crowned dens” syndrome in a 71-year-old female patient. Sagittal T1-w (**A**), STIR (**B**), and fat-suppressed contrast-enhanced T1-w (**C**) MR images showing bone marrow edema (arrows) in the C2 vertebra, abnormal paraspinal tissue, returning intermediate signals on T1-w and low on T2-w images (open arrows). Sagittal (**D**) and axial (**E**) CT images in an 85-year-old male patient with intense neck pain show to better advantage the crystal deposition (arrows).

The “crowned dens” syndrome (CDs) is a clinico-radiological entity, representing an inflammatory process resulting from crystal deposition in the ligaments surrounding the dens, with an appearance of a radiopaque “crown” surrounding the top of the dens [26]. The calcifications are mostly due to CPPD and less frequently to HADD. It typically presents with severe and acute febrile neck pain, stiffness of the cervical spine, and increased inflammatory markers. Clinically, it may mimic infectious spondylitis but also polymyalgia rheumatica, meningitis, metastatic bone disease, and primary spinal neoplasia. Clinical presentations may be asymptomatic or alarming, with acute febrile neck pain and cervical stiffness. CDs, like acute tendinitis of the longus colli, can be misdiagnosed as meningitis, infectious spondylitis, temporal arteritis, polymyalgia rheumatica, metastatic bone disease, and spinal tumor. CT shows calcifications in the ligamentous structures surrounding the top and sides of the odontoid process in a crown-like or horseshoe-like deposition (Figure 9). MR imaging may show a misleading bone marrow edema pattern.

**Figure 10:**
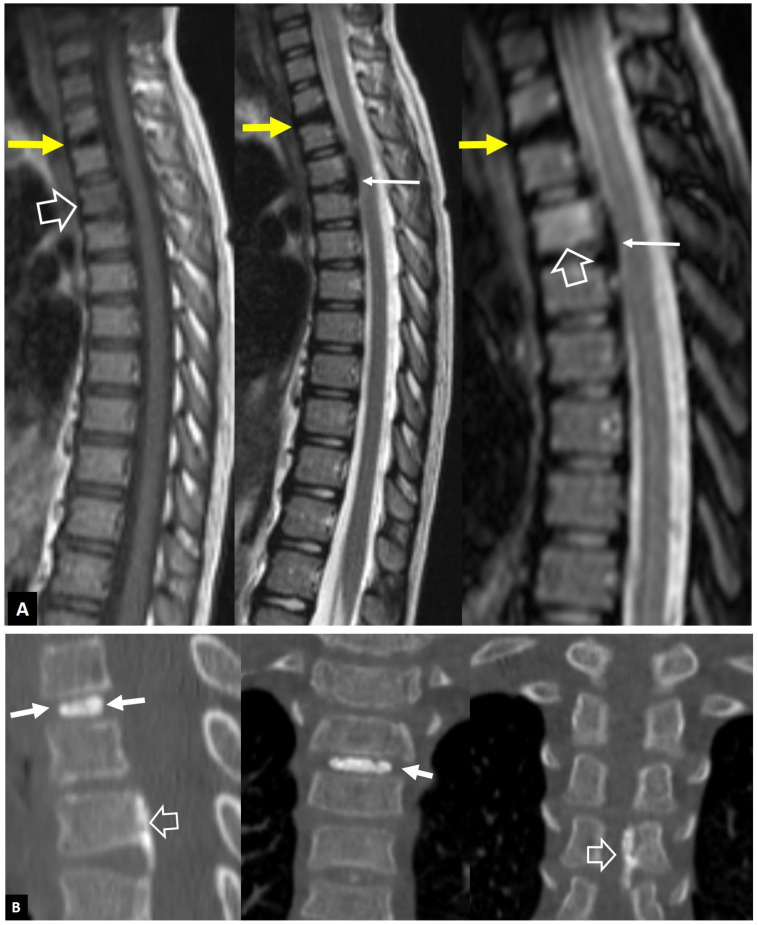
The calcification of the intervertebral disc and the ossification of the posterior longitudinal ligament in a 7-year-old girl presenting with central and left-sided neck pain for 2 weeks and with moderate alleviation with analgesics. The biochemical workup showed an ESR of 65 mm/h (normal up to 20), a CRP of 12 mg/L (normal < 5), and a normal WBC. (**A**) Sagittal T1-w (left), T2-w (center), and STIR (right) MR images showing the low signal intensity within the disc T2-T3 (arrows), the low signal and thickening of the posterior longitudinal ligament (thin arrows), and the bone marrow edema within the T4 vertebral body (open arrows). (**B**) CT reconstructions in the sagittal (left) and coronal (center, right) showing the intervertebral T2-T3 disc calcification (arrows) and the ossification of the posterior longitudinal ligament (open arrows) at the T4 vertebra and the T4–T5 intervertebral space.

A rare disorder in children is the “intervertebral disc calcification with ossification of the posterior longitudinal ligament”. The etiology is unclear, and only eight cases have been reported in the literature. An increased erythrocyte sedimentation rate (ESR) is the most valuable marker, and a complete relief of symptoms occurs within a few weeks with NSAIDs, analgesics, and a collar. A complete resolution occurs within 2 years in the majority of cases. MR imaging shows the low signal intensity of the ossified ligament but may be misleading due to the presence of bone marrow edema. CT is the method of choice for depicting disc calcification and ligamentous ossification (Figure 10) [27].

#### 2.1.6. Post-Operative Aseptic Discitis

The interpretation of the MR images of the lumbar spine must be undertaken with caution within the 6–8 postsurgical week period. Normal post-operative changes occur within the bones, the soft tissues, and the discs and vary depending on the type of surgery and the time since the operation [28].

**Figure 11:**
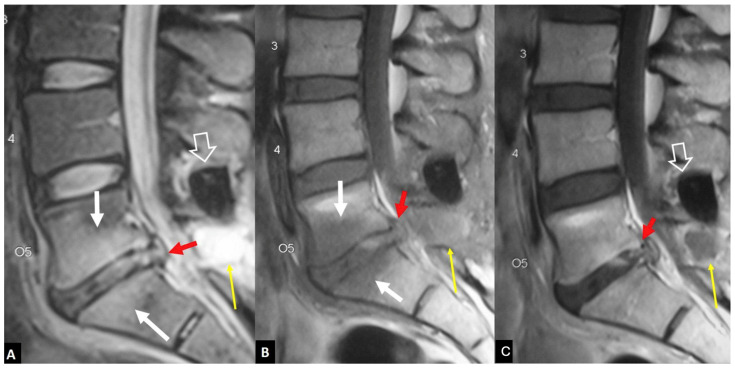
A 45-year-old male with complete resolution of symptoms following an L5 laminotomy and L5-S1 discectomy operation 6 weeks prior to imaging. Sagittal T2-w (**A**), T1-w (**B**), and contrast-enhanced T1-w (**C**) MR images showing bone marrow edema (white arrows) and L5-S1 disc with an abnormal signal and enhancement (red arrows), simulating disc herniation. A device for intervertebral assisted motion is also shown (open arrows) with a non-infectious, as proved with CT-guided aspiration, post-operative effusion (thin long arrows).

On unenhanced MR images immediately after surgery, post-discectomy changes can mimic the pre-operative appearance of disc herniation because of disruption of the annulus fibrosus and inflammatory and granulation tissue in the epidural space (Figure 11). It has been reported that, in 24% of asymptomatic patients, a residual or recurrent disc herniation is seen at the operated level within 6 weeks of surgery [28]. Bone marrow edema and enhancement at the vertebral endplates is an expected finding between 6 and 18 months after surgery in 19% of patients [29].

The intradiscal altered signal and enhancement, combined with the presence of bone marrow edema, may mimic infectious spondylodiscitis, which is a complication occurring in up to 3% of patients who have undergone non-instrumentation surgery [30].

#### 2.1.7. Trauma

**Figure 12:**
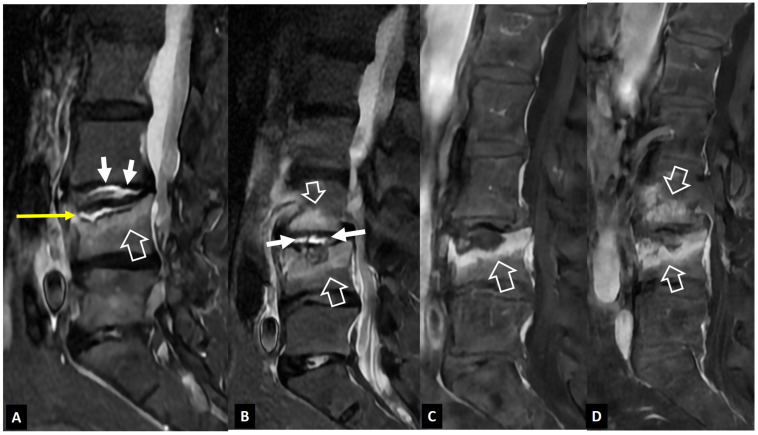
A 67-year-old female with low back pain for 3 weeks, following a minor trauma. A recent DEXA showed osteoporosis. Sagittal (**A**) and left parasagittal (**B**) STIR MR images, and corresponding fat-suppressed contrast-enhanced T1-w MR images (**C**,**D**), showing bone marrow edema on both sides of the L3–L4 disc space with enhancement (open arrows). A “fluid” sign (yellow arrow in (**A**)) and a linear delamination injury of the disc (arrows in (**A**,**B**)) do not show enhancement after contrast administration.

Acute spinal fractures, particularly in the absence of major trauma, may be a challenging clinical diagnosis. MR imaging shows bone marrow edema on both sides of the disc space, rarely with abnormal trauma-related signals within the disc (Figure 12). The absence of enhancement in the disc is a helpful sign favoring the correct diagnosis.

**Figure 13:**
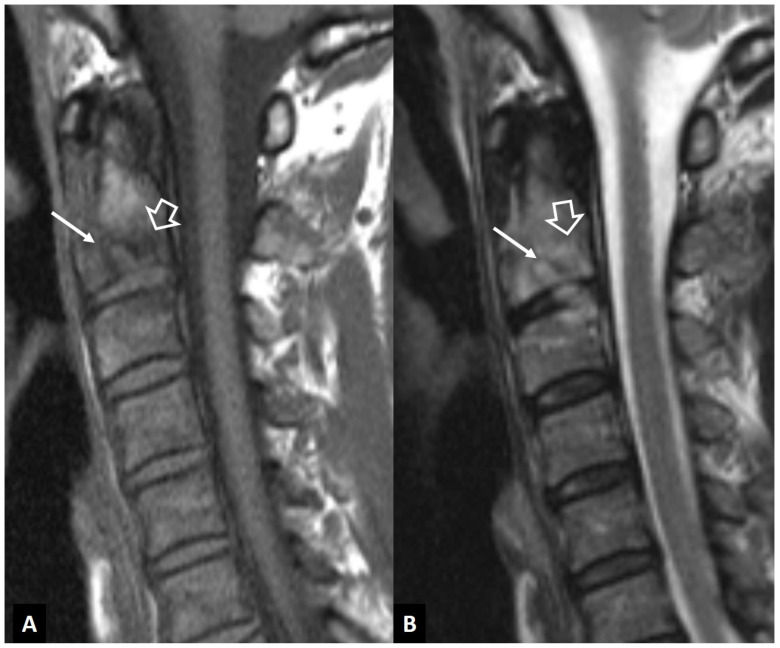
A 48-year-old male elite biker with intense pain during training. Sagittal T1-w (**A**) and STIR (**B**) MR images showing the Schmorl’s node within the C2 vertebra (thin arrows) and the surrounding bone marrow edema (open arrows). Clinical improvement was achieved with rest, painkillers, and NSAIDs over 3 weeks.

Schmorl’s nodes represent the migration of the nucleus pulposus of a disc through an insufficient or fractured endplate. The intraosseous disc protrusion is surrounded by fat-suppressed MR imaging with BME, which enhances in the acute setting in keeping with the intense clinical symptoms (Figure 13). The sclerotic ring around the migrated material allows for distinction from infectious spondylodiscitis [31].

**Figure 14:**
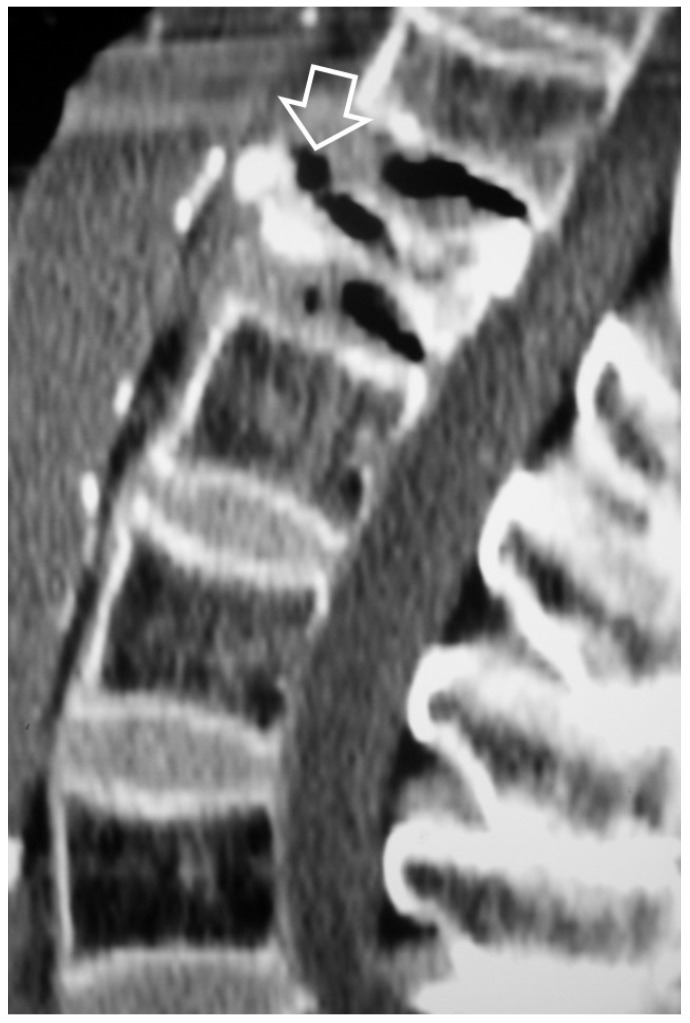
A 71-year-old female patient with an old osteoporotic fracture of the L1 vertebral body. CT sagittal reconstruction showing fragmentation and osteosclerosis, which may mimic tuberculous infection. The presence of gas within the osteonecrotic cavity under the endplate (arrow) suggests the correct diagnosis.

Chronic vertebral compression fractures may show osteosclerosis with fragmentation and global deformity. In this case, it may mimic infectious spondylodiscitis, mostly tuberculous spondylitis, if the endplate is poorly defined in the absence of an obvious history of trauma (Figure 14) [32].

#### 2.1.8. Rheumatoid Arthritis

RA of the cervical spine can mimic an infection because they share the presentation with pain, swelling, fever, elevated inflammatory markers, and neurological symptoms.

**Figure 15:**
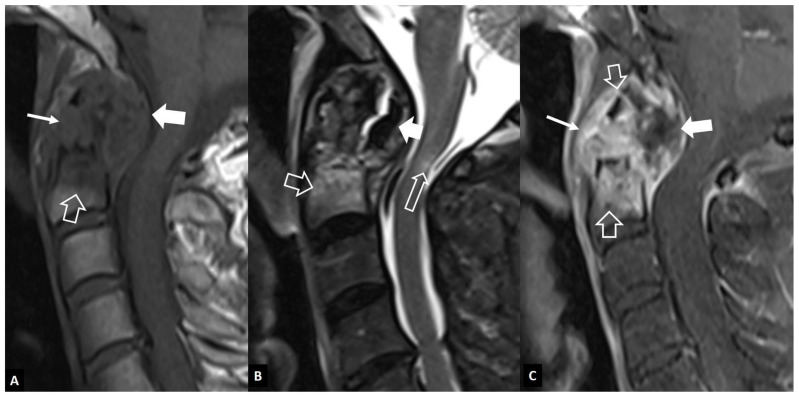
A 64-year-old male patient with long-lasting rheumatoid arthritis of the hands and wrists and progressive onset of pain, increased reflexes and numbness in the arms, and neck pain, 5 months prior to imaging. Sagittal MR images. (**A**) T1-w image showing bone marrow edema in the C2 vertebral body (open arrow), cortical disruption and soft-tissue changes in the anterior odontoid process (thin arrow), and an intermediate signal intensity mass posterior to C2 (thick arrow) compressing the spinal cord. (**B**) T2-w image showing to better advantage the bone marrow edema (open arrow), the low signal intensity mass posterior to C2 (arrow), suggesting pannus formation and a small area of edema within the spinal cord (long open arrow). (**C**) The fat-suppressed contrast-enhanced T1-w image shows the enhancement of the bone marrow edema (open arrows) and the prevertebral changes (thin arrow). The pannus tissue is only peripherally enhancing (thick arrow).

MR imaging is the most sensitive and specific imaging modality for detecting cervical spine involvement and should be performed on all patients with suspected or obvious plain radiographic anomalies or clinical evidence of myelopathy or radiculopathy [33]. MR imaging shows bone marrow edema and dens erosions, which may mimic infection, as well as soft-tissue changes including pannus formation (Figure 15). The latter corresponds to granulation tissue and hyperplastic synovium, which forms as a consequence of persistent synovial inflammation. The pannus subsequently invades the subchondral bone, resulting in the characteristic radiographic findings of erosions.

### 2.2. Non-Infectious Sacroiliitis

**Figure 16:**
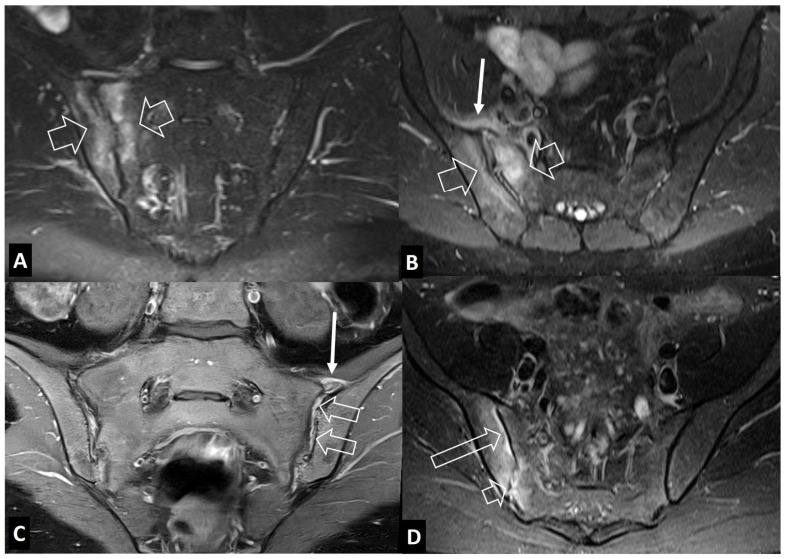
Oblique coronal (**A**) and oblique axial (**B**) STIR MR images in a 45-year-old male patient with infectious sacroiliitis showing subarticular bone marrow edema (open arrows), high intra-articular signal intensity and soft-tissue edema anterior to the joint (thin arrow). (**C**) Oblique coronal fat-suppressed contrast-enhanced T1-w MR image in a 32-year-old female patient with psoriatic axial spondyloarthropathy, showing intra-articular enhancement (open arrows) and anterior capsulitis (arrow). (**D**) Oblique axial STIR MR image in a 24-year-old female patient with a history of Familial Mediterranean fever and recent respiratory tract infections, showing subarticular bone marrow edema (long open arrow) and high intra-articular signal intensity (short open arrow), in keeping with reactive arthritis.

Infectious sacroiliitis is a relatively rare joint infection, representing under 2% of septic arthritis cases. MR imaging is the most accurate imaging modality to detect subchondral bone marrow edema, erosions, effusion in the synovial part of the joints, interosseous ligament inflammation, capsulitis, and periarticular edema (Figure 16A,B) [34]. The main differential diagnosis is axSpA, in which, apart from the subchondral bone marrow edema, capsulitis and interosseous ligament and synovitis may occur (Figure 16C) [35]. Rarely, reactive arthritis from a previous respiratory or other infection may mimic infectious sacroiliitis (Figure 16D).

The involvement of the sacroiliac joints by the crystal deposition disease and RA is rare.

## 3. Long Bones

Long bone osteomyelitis can share similar imaging features with a variety of conditions, spanning from stress injuries, benign and malignant neoplasms, and iatrogenic/radiation osseous alterations. An assessment of long bone disease should be accompanied by the evaluation of surrounding soft-tissues, which can indicate the extent of the disease. A combination of an accurate history, a laboratory examination, and imaging findings can be useful in reaching a final diagnosis. This section pertains to the mimickers of long bone osteomyelitis, and an image-based approach to the diagnosis of these conditions is discussed.

### 3.1. Stress Injuries

**Figure 17:**
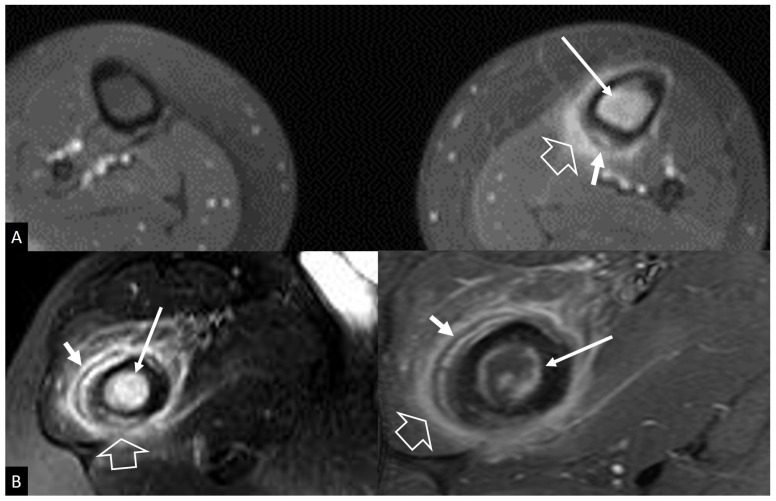
(**A**) Stress reaction in an 11-year-old boy with pain in the tibia exaggerated with football training. Axial fat-suppressed contrast-enhanced T1-w MR image, showing the bone marrow edema (long thin arrow), the soft-tissue edema (open arrow), and the periosteal reaction (short arrow). (**B**) Ewing’s sarcoma in the right proximal femoral bone in a 17-year-old male patient. Axial fat-suppressed T2-w (left) and fat-suppressed contrast-enhanced T1-w (right) MR images showing the bone marrow edema (long thin arrow), the soft-tissue edema (open arrow), and the multilayered periosteal reaction (short arrow).

A history of overuse is the key to diagnosing stress injuries. Nonetheless, when clinical information has not been provided or is obscure, the differentiation between osteomyelitis and long bone stress injuries can be difficult. Stress injuries include a spectrum of conditions starting from stress reactions (no fracture line) to complete fractures. Evaluation should always start with a plain radiograph where, in the early stages of stress injuries, the lucency of the cortex (“grey cortex” sign [36]) could be misdiagnosed as osteolysis related to infection [37]. As the injury progresses, the presence of a fracture line could indicate a stress fracture [38]. Later at the continuum of a stress injury, a solid periosteal reaction can be identified, representing callus formation, which can mimic the imaging appearance of chronic osteomyelitis. Plain radiographs have a low sensitivity for the detection of stress injuries, lower than 35% for early stress injuries, and up to 70% for the detection of a stress fracture. MR imaging is the modality of choice for the identification of a long bone stress injury. Bone marrow edema in early stress reactions can be depicted with a high signal intensity on fluid-sensitive sequences and a low signal intensity on T1-w sequences. At the stressreaction stage, distinguishing between infections and stress injuries can be completed based on the history of overuse and other clinical/laboratory characteristics. The presence of surrounding soft-tissue edema, which can also exist in the context of stress injuries, can further complicate the diagnosis. The identification of a low signal intensity fracture line within the area of edema is the key to recognizing a stress fracture (Figure 17A). The location of the lesion could also provide additional clues, since stress injuries happen in heavily mechanically loaded bones (e.g., metatarsals, tibia, and femur) and are typically located at the mid-diaphysis, whereas hematogenous osteomyelitis usually has an epicenter at the area of the bone’s vascular supply at the metaphysis [39]. Triple-phase bone scintigraphy is not useful for the differentiation between trauma and osteomyelitis with high sensitivity, except with a specificity that is less than 40%. Nonetheless, radiolabeled white blood cells can be useful and specific for the identification of underlying infection.

### 3.2. Neoplasms

#### 3.2.1. Ewing’s Sarcoma

Ewing’s sarcoma has an incidence of 2.9/10^6^ individuals in the US, ranking as the second most prevalent malignant bone tumor in children and young adults. Signs, symptoms, and laboratory findings include local swelling, pain, and an increased ESR [40]. Ewing’s sarcoma shares similar imaging characteristics to osteomyelitis, rendering the differentiation between the two entities extremely difficult (Figure 17B). A series of clinical, laboratory, and imaging factors have been found to be helpful in distinguishing between the two conditions. In terms of demographics and history, African Americans and patients with sickle cell disease are more likely to have osteomyelitis rather than Ewing’s sarcoma [41]. In terms of imaging findings, the presence of a soft-tissue mass has been shown to be more likely associated with a sarcoma. The same study showed a trend for Ewing’s to have more frequent permeative cortical disruption, even though the finding was not statistically significant, potentially due to the low sample size of the study [41].

#### 3.2.2. Lymphoma

Typical Hodgkin’s lymphoma is located in lymph nodes. Nonetheless, extranodal localization can be identified in one in five cases of the disease, involving organs such as the spleen, the bone marrow, and the liver [42]. Imaging features of extranodal lymphoma can mimic osteomyelitis when affecting the bone marrow of long bones [43]. Similarly, primary bone lymphoma, which is a non-Hodgkin lymphoma, also shares similar imaging characteristics as a long bone infection [44]. The majority of primary bone lymphoma lesions show an osteolytic (70%) or mixed lytic/blastic pattern (28%), located mostly at the meta-diaphysis or mid-shaft of the femur and tibia. An accompanying soft-tissue mass can be identified in almost half of the patients, which can be nicely seen on MR imaging, can be recognized in 80% of CT scans, and is only visible on approximately 40% of plain films [45]. It should be pointed out that, in the presence of extensive marrow lesions, associated soft-tissue masses are usually accompanied by cortical disruption in infectious conditions, but the cortex can be spared in cases of round cell tumors such as lymphomas and Ewing’s sarcomas [45]. In fact, in the early stages, plain radiographscan appear almost normal, increasing the difficulty of early diagnosis. Therefore, even with negative radiographs, in the presence of symptoms, MR imaging should be performed [46]. At the base of lytic lesions, sclerotic patterns can appear post-radiotherapy or post-chemotherapy [46]. Finally, in 1/10 cases of bone lymphoma, a sequestrum can be found that mimics chronic osteomyelitis and other tumors such as fibrosarcomas and eosinophilic granulomas [47].

### 3.3. Radiation Osteitis–Iatrogenic

Tissue damage following radiation therapy can lead to bone lysis followed by disorganized osteoblastic activity, leading to a patchy bone appearance. On MR imaging, bone marrow edema is present with a variable degree of surrounding soft-tissue edema [46]. Such radiation-induced changes are characteristic of osteonecrosis of the jaw [48]. The presence of an abscess and expansion of the bone are more indicative of infection rather than radiation-induced changes [48].

## 4. Peripheral Joints

Septic arthritis is a medical emergency secondary to an infectious agent, usually bacterial, but also due to fungal, mycobacterial, viral, or other pathogens. A delayed diagnosis and treatment may lead to rapidly progressive and irreversible joint damage and increased morbidity and mortality. Along with the heterogeneity and limited sensitivity of clinical findings, imaging appearances of septic arthritis are also non-specific, overlapping substantially with various non-infectious entities [39,49]; besides, several conditions may be superimposed on a septic joint, producing atypical clinical and imaging appearances. Especially MR imaging, despite its 100% sensitivity to showing infectious changes even at 24 h after the onset of symptoms, lacks specificity, sharing similar features with non-infectious etiologies [50]. Importantly, this may lead to diagnostic challenges during the workup of a clinically suspected septic joint or during the interpretation of imaging studies performed for other indications. Knowledge of the disorders that could resemble septic arthritis is essential for accurate diagnosis. The imaging characteristics of infectious arthritis and its potential mimics are described below with emphasis on specific imaging features aiding in distinguishing an infective from a non-infectious etiology.

### 4.1. Imaging Features of Septic Arthritis

**Figure 18:**
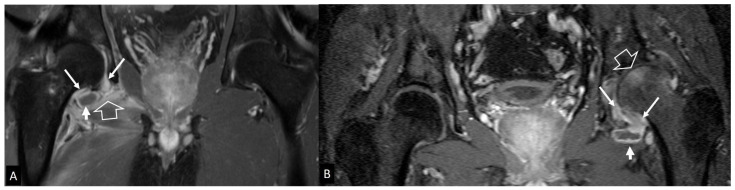
(**A**) Coronal fat-suppressed contrast-enhanced T1-w MR image in a 65-year-old male with staphylococcal septic arthritis, showing effusion (short arrow), synovitis (arrows), and soft-tissue edema (open arrow). (**B**) Coronal fat-suppressed contrast-enhanced T1-w MR image in a 59-year-old male with rheumatoid arthritis with left hip joint involvement, showing effusion (short arrow), synovitis (arrows), and bone marrow edema (open arrow).

Plain radiographs should be used for the initial evaluation of septic arthritis as they provide an overview of the anatomic area of interest and can aid in the differential diagnosis by excluding radiographically evident conditions that may clinically resemble infection [51]. Plain radiographs, although insensitive to the early changes, may reveal widened joint spaces due to joint effusion, soft-tissue bulging, and periarticular osteopenia. Advanced disease presents with non-specific erosions and uniform joint space narrowing. Radiographs cannot reliably identify joint effusions in the hips, shoulders, wrists, or small joints, where further evaluation with cross-sectional imaging is justified [52]. The presence of a joint effusion should prompt an arthrocentesis, preferably under image guidance. Ultrasound is highly sensitive for identifying joint effusion, and it can assess fluid collections in the periarticular soft tissues and guide aspiration [53]. A septic from a sterile effusion cannot be reliably differentiated based on the echogenicity of the effusion and color Doppler characteristics on the ultrasound [54]. MR imaging is not indicated as a first-line modality for the assessment of infectious arthritis due to its limited specificity [51,52]. However, the method is indicated in the context of ineffective aspiration of joint fluid or in atypical cases with contradicting imaging and clinical features. MR imaging findings suggestive of infection include synovial enhancement, perisynovial edema, joint effusion, articular cartilage destruction, bone marrow edema-like changes, and erosions of the bare areas as well as the soft-tissue involvement with potential abscess formation (Figure 18A) [55]. Tuberculous arthritis is a specific form of septic arthritis due to Mycobacterium tuberculosis. MR imaging is an important diagnostic tool for showing effusion and synovial thickening, which may assume hypointensity on T2-w images due to the presence of granuloma. Similar to pyogenic arthritis, bone erosions are present, however, typically without associated bone marrow signal abnormalities in the early disease. Periarticular abscess formation is another important feature, characterized by a thin and smooth wall and sharply defined outer borders, contrary to the thick, irregular, and nodular wall surface seen in pyogenic abscesses [56].

### 4.2. Imaging Features of Septic Arthritis Mimickers

#### 4.2.1. Inflammatory Arthritis

Among the whole spectrum of inflammatory arthritis, the main differential diagnosis for a septic joint is RA and seronegative spondyloarthropathy. From a clinical viewpoint, the onset of symptoms of inflammatory arthritis is usually subacute in contrast to the acute manifestation of septic arthritis. Notably, enthesitis, the hallmark of seronegative arthropathies, is often underdiagnosed clinically, due to the low sensitivity and specificity of clinical tests. Different from the monoarticular pattern of septic arthritis, RA typically shows a polyarticular distribution involving the small joints of the upper and lower extremities, whereas seronegative spondyloarthropathy has a typical axial predominance besides showing a predilection mainly for lower extremity peripheral joints. The elevation of inflammatory markers including ESR and CRP are frequently seen in both inflammatory and infectious entities. A specific serological workup should be pursued when autoimmune inflammatory arthritis is in the differential. Radiographs are able to detect structural joint damage in established diseases but are insensitive to depicting early soft-tissue or bone changes [57]. MR imaging and ultrasonography are the methods of choice for evaluating RA. Early findings include synovitis showing a thick enhancement pattern following contrast administration, joint effusion, and marginal, bare-area erosions (Figure 18B) [58]. Hypertrophic synovial villi may detach into the joint space, forming fibrinous nodules termed “rice bodies” [58]. Bone marrow edema is commonly seen in subchondral locations or surrounding erosions but is less extended compared to infectious arthritis [59]. Ultrasound typically shows synovitis as thickened and hypoechoic intra-articular tissue, usually together with variable amounts of anechoic joint effusion. Bone erosions are seen as intra-articular bone surface discontinuities in two perpendicular planes. A high Doppler signal suggests the presence of hypervascularized pannus tissue in active erosion [60]. The combination of bone erosions with extensive marrow changes, the presence of perisynovial edema, and soft-tissue abscesses favor the diagnosis of joint infection over RA [58]. Enthesitis, defined as inflammation at tendinous, ligamentous, and capsular attachments, together with bony proliferations, are the hallmarks of seronegative arthropathies, serving as discriminating features between seronegative and other types of inflammatory arthritis [61]. Both ultrasound and MR imaging are highly specific in detecting acute and chronic enthesis-centered abnormalities [61]. Ultrasound features include decreased echogenicity, thickening, calcification, and increased Doppler vascularity of the affected structure. Erosions or enthesophytes are indicated by osseous surface abnormalities, while increased Doppler synovial vascularity suggests synovitis. Bone marrow edema at an enthesis, but not only confined to it, and perientheseal soft-tissue changes represent the hallmark of active enthesitis on MR imaging. Chronic enthesitis is characterized by erosions or enthesophytes best seen on T1-w sequences. Additionally, screening of the axial skeleton for typical disease manifestations in equivocal cases may be of value.

#### 4.2.2. Neuropathic Arthropathy

Neuropathic (or Charcot) arthropathy refers to a progressive, destructive joint disorder occurring in patients with abnormal pain sensations and proprioception, most commonly due to underlying diabetes mellitus but also rarely due to other sensorimotor and autonomic neuropathies of various etiologies. The foot joints are by far the most commonly affected, although the knee, wrist, and spine may rarely be involved. Joint swelling, warmth, and erythema, regularly preceding the radiographic changes, may resemble infectious or inflammatory arthritis in early disease stages [62]. More importantly, infection is often superimposed on neuropathic disease, posing challenges to the distinction between the two abnormalities [62]. Radiographs have a fundamental role in diagnosis and disease monitoring, while MR imaging is essential for diagnosing early stages, detecting complications, including superimposed infections, and assessing the disease extent [63]. Based on radiographic and clinical features, neuropathic arthropathy is divided into three stages: (i) development, characterized by osteopenia, periarticular debris/fragmentation and joint subluxation/dislocation; (ii) coalescence, showing the absorption of periarticular debris, early sclerosis, and the fusion of the large bony fragments; and (iii) reconstruction, characterized by new bone formation and joint arthrosis/ankylosis [64]. The MR imaging features of a neuropathic joint, including diffuse soft-tissue edema, joint effusion, periarticular fluid collections, and bone marrow abnormalities gradually proceeding to joint derangement, may be misinterpreted as an infectious process, which is further complicated by the inflammatory clinical profile typical for early disease stages. Useful discriminating features pointing toward infectious over neuropathic arthritis include diffuse or thick peripheral instead of thin rim synovial enhancement, less prominent osseous debris, the relative absence of subchondral cystic lesions being almost exclusively seen in non-infected joints, the loss of subcutaneous fat signal intensity adjacent to the joint on T1-w sequences, although soft-tissue edema is seen with similar frequencies around both infected and neuropathic joints and the presence of a sinus tract in the periarticular soft tissue. Additionally, especially for the foot, diffuse bonemarrow edema, involving the entire bone, is significantly more common in the setting of osteomyelitis instead of neuropathic-related changes [64].

#### 4.2.3. Crystal-Induced Arthropathies

**Figure 19:**
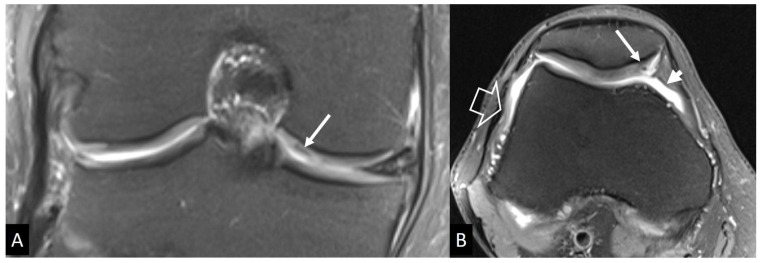
A 62-year-old athletic male, with severe joint pain, warmth, and swelling. The final diagnosis was CPPD arthritis. Coronal (**A**) and axial (**B**) fat-suppressed PD-w MR images showing mild effusion (short arrow). Synovitis (open arrow) and chondrocalcinosis (thin arrows) suggest CPPD.

**Figure 20:**
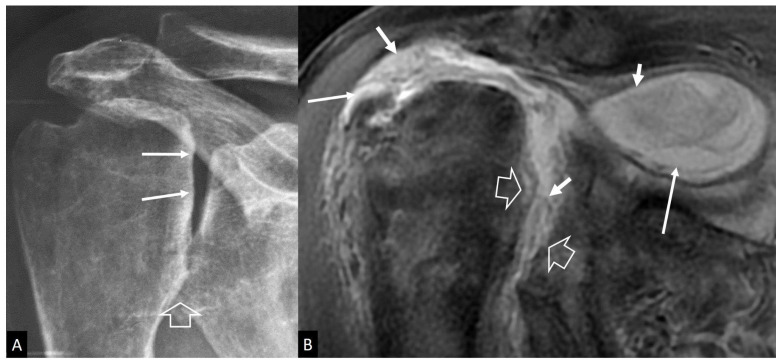
A 77-year-old male patient with mild pain, reduced range of motion, crepitation, and joint instability lasting 1 year prior to imaging. (**A**) Characteristic radiographic features of “Milwaukee” syndrome showing loss of the round configuration of the humeral head (arrows) and joint space narrowing at the inferior glenohumeral joint (open arrow). (**B**) Coronal oblique, fat-suppressed PD-w MR image showing massive rotator cuff tear, large joint effusion extending to bursae (long arrows), internal debris and synovial proliferation (short arrows), and subchondral bone and cartilage destruction (open arrows).

Crystal-induced arthropathies, especially in the context of CPPD and HADD, are common conditions characterized by synovial and periarticular crystal deposition, which may lead to variable clinical manifestations, ranging from asymptomatic to acute arthritis and chronic arthritis or destructive arthropathy [65]. In the acute phase of CPPD, the symptoms of joint edema, erythema, tenderness, and low-grade fever, in up to 50% of patients may simulate inflammatory or infectious processes [66]. The knee joint is most commonly affected, followed by the shoulder, wrist, metacarpophalangeal, and hip joints. A subset of patients with CPPD may also experience waxing and waning episodes of non-synchronous inflammatory arthritis affecting multiple non-weight-bearing joints, such as wrists and metacarpophalangeal joints [67]. In the diagnostic assessment of CPPD, conventional radiography and ultrasound, or CT if axial involvement is suspected, are the recommended imaging modalities [68]. Plain radiographs typically show chondrocalcinosis, as the hallmark of the disease, appearing as punctate and linear densities in the hyaline cartilage/fibrocartilage. The triangular cartilage of the wrist, the fibrocartilage of the pubic symphysis, and the menisci are the most common locations. Synovium and tendinous calcifications may coexist, depicted as thin linear densities along the synovial membrane or the length of tendons. Other radiographic findings, which are of value, especially in the absence of chondrocalcinosis, include features of osteoarthritis, primarily demonstrating joint space narrowing, geodes, and the relative absence of osteophytes. Ultrasound is able to detect chondrocalcinosis, mainly following three distinct patterns: (i) thin hyperechoic bands paralleling the articular cartilage surface; (ii) punctate hyperechoic bands in regions of fibrocartilage; and (iii) homogeneous, hyperechoic intra-articular nodular or oval deposits likely representing free crystal aggregates [69]. CT shows chondrocalcinosis to better advantage and is of value in equivocal cases or in axial involvement [70]. MR imaging is of limited value, although, particularly with the use of gradient-echo sequences for the evaluation of crystal deposition, they may be of diagnostic aid (Figure 19) [70]. HADD is associated with two important musculoskeletal syndromes. The deposition of crystals along periarticular soft tissues causes calcific periarthritis, whereas intra-articular deposition contributes to a highly destructive shoulder-predominant arthritis known as “Milwaukee” syndrome. Periarticular deposition can be easily diagnosed with radiographs and ultrasound and does not cause diagnostic dilemmas [68]. Imaging features of “Milwaukee” syndrome overlap with advanced osteoarthritis, neuropathic, and septic arthropathy. Contrary to infectious arthritis, clinical symptoms are usually mild compared to the radiological progression, usually including a large glenohumeral joint effusion, crepitation, and joint instability. Characteristic radiographic features include marked glenohumeral joint degeneration with intra-articular loose bodies, soft-tissue calcifications, and extensive rotator cuff damage manifesting as a “high-riding” humeral head. MR imaging and ultrasound can reveal large joint effusion with internal debris and synovial proliferation, joint narrowing with subchondral bone, and cartilage destruction together with advanced rotator cuff tears (Figure 20).

#### 4.2.4. Rapidly Destructive Osteoarthritis of the Hip

**Figure 21:**
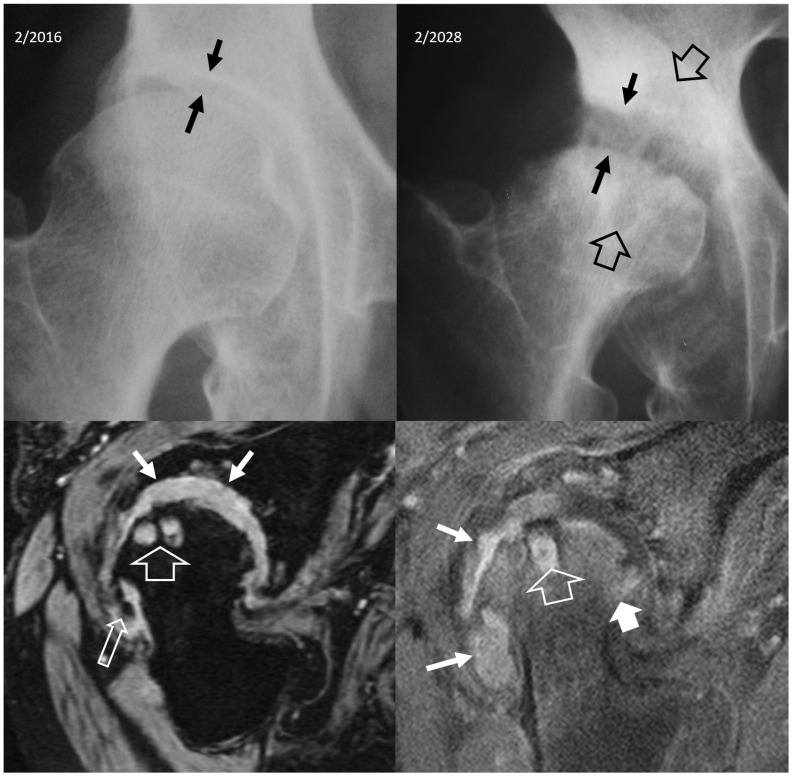
Rapidly destructive osteoarthritis of the hip in a 72-year-old female patient with constant pain and limited range of motion. A plain radiograph (upper left) taken with the onset of symptoms shows a normal joint space (arrows). Two years later, a plain radiograph shows the widening of the joint space (arrows) and subarticular cyst formation (open arrows). Oblique axial gradient echo (lower left) and fat-suppressed contrast-enhanced T1-w (lower right) MR images showing subarticular cysts (open arrows), osteophyte formation (thin open arrow), synovitis (arrows), and bone marrow edema (thick arrow).

Being almost specific to the hip joint, rapidly destructive osteoarthritis of the hip (RDOH) is characterized by a clinical history of hip pain lasting 1–6 months and radiographic features of rapidly progressive bone destruction involving the femoral head and acetabulum within months of the onset of symptoms, in the absence of clinical or laboratory evidence of an infectious, neurologic, metabolic, or inflammatory background. The disease was first described as a chondrolysis of >2 mm in 1 year or 50% joint space narrowing in 1 year [71]. Typically, radiographs show joint-space narrowing, usually located superolaterally, minimal subchondral sclerosis, and a relative absence of osteophytes [72]. The key early MR imaging features of RDOH include a significant loss of articular cartilage as compared to the contralateral side, rarely widening, synovitis/joint effusion, bone marrow edema in the femoral head/neck and/or the acetabulum, being most prominent at the weight-bearing areas, cyst-like subchondral defects, and subchondral insufficiency fractures of the femoral head (Figure 21). The latter appears as a curvilinear low signal intensity band on all sequences, which parallels the overlying subchondral bone. Focal signal abnormalities in the adjacent soft tissues are present in one-third of cases, potentially causing diagnostic confusion with septic arthritis [73,74]. In the late stage, radiographs are diagnostic, showing femoral head flattening and superior-lateral joint-space narrowing often mimicking neuropathic arthropathy, without necessitating further imaging evaluation. Useful discriminatory features from septic arthritis are subchondral geodes, the femoral head deformity usually being disproportionately advanced compared to the acetabular derangement, and the relatively uncommon presence of soft-tissue involvement without abscess formation.

#### 4.2.5. Neoplasms

**Figure 22:**
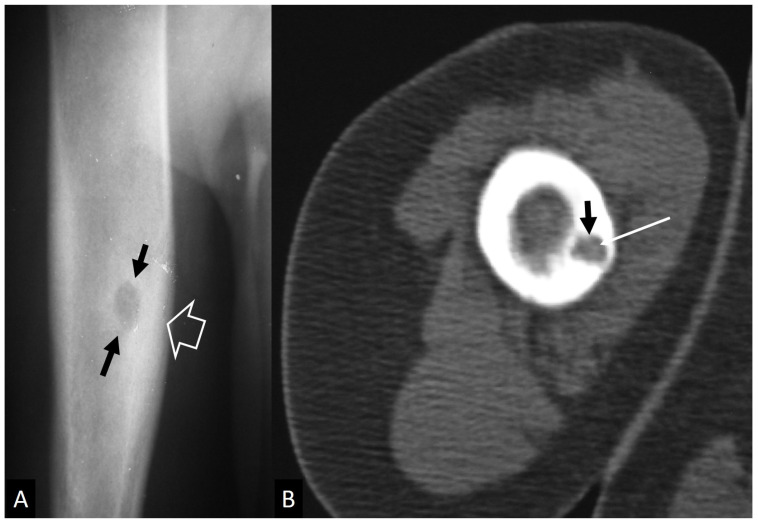
An 11-year-old girl with a surgically proven Brodie’s abscess. (**A**) The plain radiograph shows the lytic lesion (arrows) and the surrounding sclerosis (open arrow). (**B**) Axial CT image showing the lytic lesion (arrow) and a central hyperdensity simulating a nidus of osteoid osteoma (thin arrow).

Osteoid osteoma represents a specific neoplasm that could be confused with infection. Although predominantly affecting the diaphysis of long bones, osteoid osteoma can also assume an intra-articular location, most commonly around the hip joint. Intra-articular prostaglandins’ secretion promotes lymphoproliferative synovitis, leading to atypical clinical symptoms of pain, stiffness, and high local temperature, potentially mimicking inflammatory or infectious arthritis [75]. Additionally, the lack of the typical nocturnal worsening and poor improvement with NSAIDs further complicate the clinical assessment of intra-articular lesions. Plain radiographs have low sensitivity in diagnosing intra-articular osteoid osteomas because of the minimal periosteal new bone formation in these forms, in contrast to the florid periosteal reaction typically seen in their extra-articular counterparts [76]. This is due to the limited periosteal apposition within the joint related to the absence of the internal layer of the periosteum. Intra-articular lesions have distinct features in MR imaging, including synovitis, joint effusion, and surrounding bone marrow edema, which may be misinterpreted as an infectious or inflammatory process. The clue to the correct diagnosis is the detection of a nidus appearing usually as a T1-w hypointense and T2-w hyperintense focus surrounded by bone marrow edema; however, it may be invisible on MR imaging [77]. CT remains the standard for identifying the nidus, as a focally well-defined lucent area potentially containing a central sclerotic dot. CT may be thought inadequate for visualizing the nidus in some cases, requiring further evaluation with bone scintigraphy or fluorine 18–labeled sodium fluoride PET/CT for a definitive diagnosis [77]. Finally, especially applying to long bones, Brodie’s abscess is the main differential diagnosis for osteoid osteoma, presenting with both clinical and radiological similarities. Radiographically, Brodie’s abscess appears as a radiolucent, well-demarcated eccentric lesion with marginal sclerosis and variable periosteal thickening, while a sinus tract can be visualized especially on CT (Figure 22) [78]. A useful discriminating feature on MR imaging, being specific for Brodie’s abscess, is the “penumbra sign” referring to a discrete zone of the transition of relative hyperintensity between the intermediate to low signal abscess cavity and the adjacent bone marrow on unenhanced T1-w images [78].

## 5. Muscles and Soft Tissues

Soft-tissue and muscle infections encompass a wide range of different pathological situations, which can resemble many other conditions of different origins, such as inflammatory, neoplastic, and traumatic. Awareness of helpful imaging and clinical considerations may lead promptly to the correct differential diagnosis in such a situation, which is important due to different morbidity and mortality rates and special therapeutic management [79]. Herein, a useful radiological approach for establishing an image-based diagnosis of soft-tissue infection mimickers in combination with the appropriate history and laboratory examination is provided.

### 5.1. Non-Infectious Subcutaneous Edema

A great mimicker of cellulitis is subcutaneous edema due to non-infectious causes such as congestive heart failure, diabetic vascular insufficiency, lymphatic obstruction, and acute and subacute venous thrombosis. In addition to the absence of an infective agent, one supporting imaging feature of this type of edema is the more diffuse nature, without contrast enhancement, after intravenous administration due to non-hyperemic soft tissue in such conditions [80].

### 5.2. Non-Infectious Fasciitis

This group of diseases includes separate pathological entities like paraneoplastic fasciitis, eosinophilic fasciitis, and nodular and proliferative fasciitis, which require distinct clinicopathologic correlations to reach the correct diagnosis.

#### 5.2.1. Eosinophilic Fasciitis

**Figure 23:**
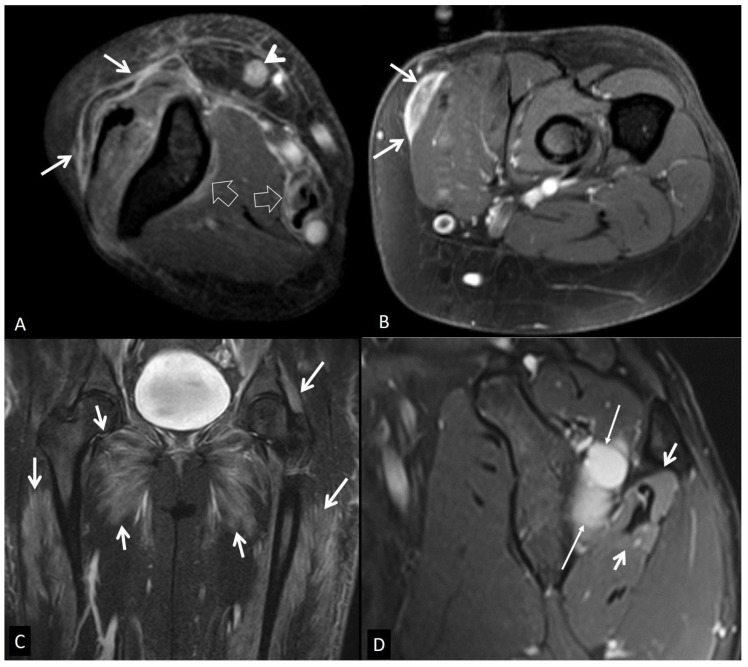
(**A**) Eosinophilic fasciitis in a 68-year-old female. Axial fat-suppressed contrast-enhanced T1-w MR images show enhancement of the superficial fasciae (arrows), deep fasciae (open arrows), and a small lymph node (arrowhead). (**B**) Nodular fasciitis in a 60-year-old female patient. Axial fat-suppressed contrast-enhanced T1-w MR image showing enhancement of the fascia (arrows). (**C**) Coronal STIR MR image in a 20-year-old female patient with dermatomyositis, showing diffuse muscle edema (arrows). (**D**) Muscle denervation in a 25-year-old male patient. Oblique sagittal PD-w MR image showing the ganglion cyst (thin arrows) and the edematous infraspinatus muscle (arrows). The rest of the rotator cuff muscles show normal signals.

Peripheral eosinophilia, hypergammaglobulinemia, increased ESR, and scleroderma-like skin changes favor a diagnosis of eosinophilic fasciitis. However, useful MR imaging findings include fascial thickening at T1-w images with corresponding hyperintense signal on fluid-sensitive sequences and a variable degree of enhancement on postcontrast images, without relative muscle belly involvement (Figure 23A) [81].

#### 5.2.2. Paraneoplastic Fasciitis

With the coexistence of a known malignancy, paraneoplastic fasciitis may occur as a rare manifestation of acute febrile neutrophilic dermatosis (“Sweet” syndrome). Non-specific findings during MR imaging include dermal thickening, soft-tissue edema in multiple sites, fascial thickening, and concomitant muscle edema in keeping with certain clinical manifestations (painful red or purple-red papules or nodules in the skin, elevated ESR, and neutrophil-predominant peripheral leukocytosis) [82].

#### 5.2.3. Nodular Fasciitis

Infiltrative fasciitis, pseudosarcomatous fasciitis, and pseudosarcomatous fibromatosis are other names of this condition, which typically affects patients younger than 50 years of age and is manifested with a rapidly growing nodule or nodules, usually less than 2 cm and in a subcutaneous, intrafascial, and intramuscular location [83]. Many different T1-w and T2-w imaging characteristics can be seen in MR imaging due to the different content of these nodules (fibrous, cellular, or myxoid), but most of the time, they show homogeneous enhancement (Figure 23B) [84].

#### 5.2.4. Inflammatory Myopathies

Autoimmune myositis is a rare group of autoimmune-associated muscle disorders, also known as idiopathic inflammatory myopathies, which recently have been classified into four major subgroups: dermatomyositis, inclusion body myositis, immune-mediated necrotizing myopathy, and antisynthetase syndrome [85,86]. Their clinical manifestations have a similar pattern involving the musculature of the thighs and pelvis or other proximal symmetrical musculature, not limited to specific compartmental or neural anatomy.

All of them share common MR imaging findings consisting of high signal intensity in fluid-sensitive sequences of muscles or perimuscular soft-tissue, with varying degrees of postcontrast enhancement (Figure 23C). In the later stages of the disease, fatty atrophy of the muscle is established, best shown with T1-w sequences [87].

### 5.3. Muscle Denervation

Muscle denervation can be observed in almost any voluntary muscle in the body and results from the partial or complete loss of innervation due to several causes, like outer nerve compression (i.e., ganglion cyst, vascular aneurysm), trauma, neuropathies, neoplasia, vasculitis, and thrombosis or compression of the draining veins. A clinical examination in combination with MR imaging and electromyography are the hallmarks of correct and verified diagnosis [88]. MR imaging changes depend on the phase of the denervation. In the acute phase, MR imaging can be normal but may show intramuscular edema within 24 hours. After 2–4 weeks of the insult (subacute phase), the affected muscle shows uniform edema and paradoxical enlargement due to either pseudohypertrophy or true hypertrophy of the muscles, shown with diffuse T2-w hyperintense signals and normal T1-w intensity signals of the muscles, while subcutaneous tissue appear normal (Figure 23D). At the subacute phase, mixed muscle edema and atrophy occur, followed by muscle atrophy in the chronic form over months [89].

### 5.4. Traumatic Soft Tissue Injury

Under the term “muscle injury”, many different conditions can be included, such as laceration, contusion, strain, muscle hemorrhage/hematoma, compartment syndrome, myositis ossificans, or exercise-related conditions, which may resemble a muscular infection. The clinical history plays a crucial role in differentiating infection from trauma-related muscular injury during MR imaging.

#### 5.4.1. Muscle Contusion

**Figure 24:**
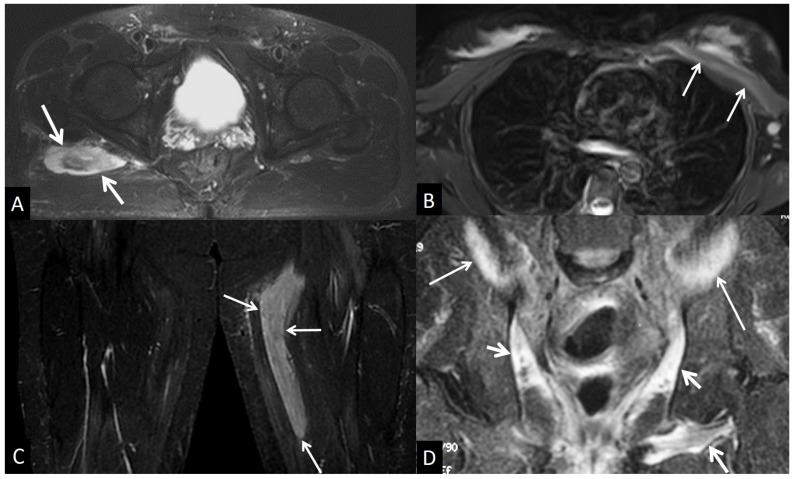
(**A**) Muscle contusion in a 65-year-old male following a fall from 2.5 m. Axial fat-suppressed T2-w MR image showing the intramuscular hematoma (arrows). (**B**) Axial fat-suppressed T2-w MR image showing the pectoralis major muscle strain (arrows). (**C**) Coronal STIR MR image showing muscular edema (arrows) in a 43-year-old female marathon runner with delayed muscle soreness syndrome. (**D**) Coronal STIR MR image in a 65-year-old male patient with bladder carcinoma, showing post-radiation changes in the iliac (thin arrows) and obturator (short arrows) muscles.

A muscle contusion is characterized by damage and/or rupture of muscle fibers due to direct impact and may cause interstitial hemorrhage and edema at the site of injury and in the underlying muscle. The MR signal intensity of the hematoma depends on the age of the blood products (Figure 24A). Subacute blood shows high T1-w signal intensity due to methemoglobin magnetic properties and chronic blood breakdown, and hemosiderin deposition shows a low-signal-intensity rim with all pulse sequences [90]. In the absence of trauma, possible anticoagulation therapy should be considered, or underlying neoplasm should be suspected.

#### 5.4.2. Muscle Strain

A muscle strain is usually confined to the myotendinous or myofascial units and frequently occurs in muscles that cross two joints, such as the hamstring, gastrocnemius, and biceps brachii muscles. The MR imaging-revealed edema is centered at the myotendinous junction and, in the early stage, is shown with a T2-w high signal with a feathery appearance (usually centered on the myotendinous junction) representing edema (Figure 24B). In the latter stages, additional features include partial or complete fiber disruption characterized by a high T2-w signal and local distortion [90].

#### 5.4.3. Delayed Onset Muscle Soreness

Delayed onset muscle soreness refers to a specific overuse muscle injury, where the symptoms are delayed by hours to days, which is in contrast to a muscle contusion or strain, where pain occurs immediately. In this reversible microstructural muscle injury, the affected muscles are high signal on fluid-sensitive MR sequences, and this may persist for months following the resolution of symptoms (Figure 24C).

#### 5.4.4. Rhabdomyolysis

Another cause of diffuse muscle edema is rhabdomyolysis, which can be classified into traumatic (e.g., crush injury, excessive exercise) and non-traumatic (e.g., extreme body temperature change, arterial occlusion, metabolic disturbances, toxins, inflammation, infection) causes. Rhabdomyolysis describes the rapid breakdown of muscle fibers with the release of intracellular contents into the systemic circulation. MR imaging show edema in the involved muscles, depicted as homogeneous or heterogeneous hyperintensity on T2-w imaging, without involving the adjacent fasciae. Depending on the severity of the disease, contrast enhancement varies between homogeneous in viable muscle and rim-enhanced in myonecrosis [91].

#### 5.4.5. Myositis Ossificans

Myositis ossificans is an extraosseous bone formation usually as a result of trauma; however, it can occur spontaneously or in other conditions such as paralysis, burns, and intramuscular hematomas. MR imaging appearances vary with the age of the lesion. At an early stage, the involved muscle demonstrates heterogeneous edema, and over a period of 6–8 weeks, a characteristic peripheral calcification develops with corresponding low signal intensity with all MR sequences [90].

### 5.5. Neoplasm and Post-Therapy Soft-Tissue Changes

#### 5.5.1. Neoplasms

A challenging differential diagnosis of muscular infection is soft-tissue and muscle edema caused by adjacent neoplasms, which is shown in MR imaging with high signal intensity on fluid-sensitive sequences, due to tumor invasion, edema, or both. Although it is necessary to distinguish between them for surgical planning and radiation therapy, most of the time, there are no reliable MR imaging findings to distinguish between edema and tumor invasion. A favorable sign of tumor invasion is the indistinct border between the muscle and the tumor [92].

#### 5.5.2. Post-Therapy Soft-Tissue Changes

Post-radiation changes in soft tissues are mostly faced after adjuvant or neoadjuvant therapy of high-grade sarcomas. Typical MR imaging findings include diffuse muscle edema with sharp and straight margins delineating the radiation field, subcutaneous lattice-like edema with overlying skin thickening, and moderate muscle enhancement after contrast media administration (Figure 24D) [90,93,94].

## 6. Conclusions

Musculoskeletal infections are challenging to diagnose accurately due to their ability to mimic a wide range of other conditions on imaging. These infections can present with imaging features that overlap with various non-infectious pathologies, such as tumors, inflammatory conditions, and trauma-related changes. As a result, radiologists must maintain a high index of suspicion and consider a broad differential diagnosis when interpreting imaging findings in cases of suspected musculoskeletal infection.

Advanced imaging techniques, such as MR imaging, play a crucial role in detecting and characterizing these infections, offering detailed insights into soft-tissue involvement, bone marrow changes, and the presence of abscesses or sinus tracts. However, the interpretation of these images requires a thorough understanding of the disease’s clinical context and an awareness of its potential to mimic other conditions.

Ultimately, the accurate diagnosis of musculoskeletal infections relies on a combination of imaging findings, clinical presentation, and, when necessary, laboratory and microbiological data. The early and precise identification of these infections is vital to initiating the appropriate treatment, preventing complications, and improving patient outcomes. Radiologists must, therefore, be vigilant and adopt a systematic approach to differentiating musculoskeletal infections from other mimicking conditions on imaging.
